# Derivation and Application of Molecular Signatures to Prostate Cancer: Opportunities and Challenges

**DOI:** 10.3390/cancers13030495

**Published:** 2021-01-28

**Authors:** Dimitrios Doultsinos, Ian G. Mills

**Affiliations:** 1 Nuffield Department of Surgical Sciences, John Radcliffe Hospital, University of Oxford, Oxford OX3 9DU, UK; ian.mills@nds.ox.ac.uk; 2 Patrick G Johnston Centre for Cancer Research, Queen’s University of Belfast, Belfast BT9 7AE, UK

**Keywords:** signatures, transcription factors, computational biology, prostate cancer, chromatin, biomarkers, prognostication, stratification, biobanking, epigenetics

## Abstract

**Simple Summary:**

Prostate cancer continues to exert a significant public health burden across the globe with hundreds of thousands of new diagnoses per year. There have been many advances in prostate cancer treatment that have dramatically improved the outlook for a lot of patients, especially by targeting a key factor in prostate cancer development called the androgen receptor. However, with increasing of targeted therapies we see a shift in the spectrum of treatment resistance disease. Molecular signatures are essentially maps of the potential for tumor evolution. By analyzing patient and pre-clinical model derived data, we may put together lists of genetic determinants of cancer progression and predict if patients will be prone to develop aggressive disease. In this manuscript we are reviewing some of the ways that these signatures are generated and discuss the advantages and disadvantages of their utility in personalized medicine.

**Abstract:**

Prostate cancer is a high-incidence cancer that requires improved patient stratification to ensure accurate predictions of risk and treatment response. Due to the significant contributions of transcription factors and epigenetic regulators to prostate cancer progression, there has been considerable progress made in developing gene signatures that may achieve this. Some of these are aligned to activities of key drivers such as the androgen receptor, whilst others are more agnostic. In this review, we present an overview of these signatures, the strategies for their derivation, and future perspectives on their continued development and evolution.

## 1. Introduction

“Cancer burden continues to grow globally, exerting tremendous physical, emotional and financial strain on individuals, families and health systems.” This excerpt from the current World Health Organization information sheet on cancer serves as a reminder that despite the huge advances in cancer-related treatments over the past few decades, cancer remains the second leading cause of death globally, accounting for roughly 10 million deaths in 2018.

The ever-increasing integration of basic scientific research into the discovery and management of new therapies is driving a shift from a population approach to a personalized approach to cancer treatment. This is supported by the accumulation and analysis of a huge amount of data derived from genomic studies that include the human genome project and the cancer genome atlas. In this review, we provide a comprehensive overview of the biology involved in transcription factor-mediated prostate pathophysiology and the tools that have led to several transcriptional signatures in clinical or research use whilst exploring the utility of such signatures.

### 1.1. Prostate Cancer

Prostate cancer (PCa) is a high-incidence malignancy that results in 360,000 deaths annually and is the second most common cause of death amongst men despite significant advances in its treatment [1]. The biological master regulator and transcription factor identified as a key player in PCa pathophysiology is the androgen receptor (AR) [2]. Targeting the AR has drastically improved the prognosis of PCa patients, but a subset of tumors can become AR-indifferent and hence resistant to AR-targeting therapies, thus leading to fatalities through metastatic diseases that are treatment-resistant [3]. This partly reflects the fact that other transcription factors and chromatin regulators may modify or substitute for some aspects of AR function. Some of these factors such as HIF1α are combinatorial targets alongside the AR and have been investigated in this capacity [4]; hypoxia, for example, induces a particular repressive transcriptional response that points towards chromatin and histone modifications affecting carcinogenesis [5].

### 1.2. Transcription Factors and Activity Signatures

Transcription factors (TFs) bind onto specific DNA sequences to regulate gene expression. TFs are regularly altered in expression and activity in various cancer types as a result of mutations, amplifications or deletions, chromosomal abnormalities, or chromatin landscape remodeling [6]. Cancer is regulated by the function or deregulation of proto-oncogenes and tumor suppressors that govern 19 signal transduction pathways that exert downstream signals through TFs managing metabolic and mutational stress and demand [7]. Considering that cancer is the result of dysregulation and miscommunication of crosstalk between key biological processes such as growth, it follows that it shares key similarities with embryological and developmental processes: pathways that are heavily regulated by TF activity [8].

Because TFs regulate target gene expression, target gene expression can be consequently used as a proxy for TF activity [9] and the combination of several target gene expression levels or activities can constitute TF activity signatures. The purpose of activity signatures in translational oncology is arguably twofold: firstly to provide a proxy for the contribution of a TF or epigenetic regulator to tumorigenesis and secondly to assess the contribution or phenotype of a hallmark of cancer [10] to tumorigenesis, be that proliferation, differentiation/de-differentiation/stemness, or others. It thus follows that the most powerful signatures will represent elements of as many of these aspects as possible and the least powerful/most reductionist will focus on just one, such as proliferation. There have been several attempts to generate TF-centered or TF-related activity signatures as prognostic or diagnostic tools with varying success. Thus, in this review, we focus on the generation and utility of transcriptional signatures in PCa.

## 2. Transcription Factors in Prostate Cancer

**Figure 1:**
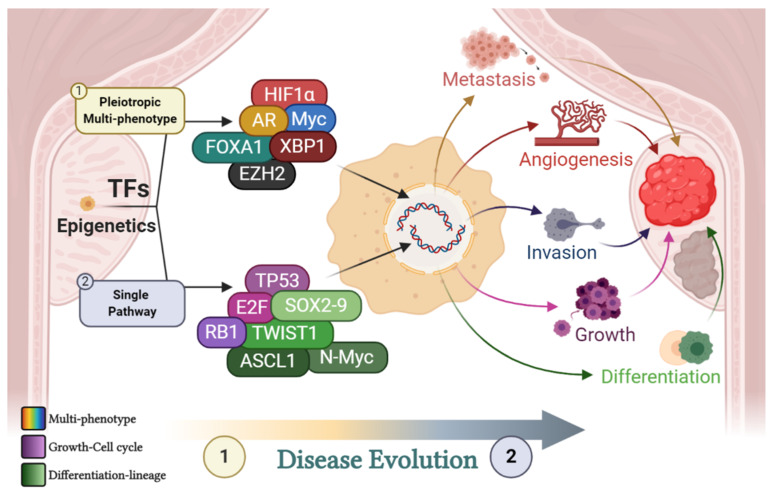
Contribution of transcription factors (TFs) to disease progression. Multiple transcription factors and epigenetic modifiers contribute to disease progression. Some are pleiotropic and affect multiple key biological pathways (multi-colored). Others (although affecting several systems in a global way) predominantly affect single key developmental and biochemical pathways such as growth (purple array) and lineage determination or differentiation (green array). Moreover, pleiotropic TFs may be predominantly active at an early stage in disease evolution, whilst single pathway TFs are most prominent later in disease progression. This is exemplified by the phenomenon that localized prostate cancer (PCa) does not display a high proliferative index or huge fluctuations in the prostate-specific antigen (PSA) level prior to treatment (a marker of a transformed, differentiated prostate). As such, TFs are ever-present throughout disease evolution and collaborate to promote tumor differentiation, growth, angiogenesis, invasion, and metastasis. The purple array includes tumour protein 53 (TP53), E2 factor (E2F) and retinoblastoma protein (RB1). The green array includes the twist family BHLH transcription factor 1 (TWIST1), achaete-scute family BHLH transcription factor 1 (ASCL1), N-Myc and the sex determining region Y-box 2 (SOX) TF family. Individual TFs include the androgen receptor (AR), enhancer of zeste homologue 2 (EZH2), x-box binding protein 1 (XBP1), forkhead box A1 (FOXA1), Myc and hypoxia inducible factor 1 (HIF1alpha). Created with BioRender.com.

As introduced in the previous section, TFs and epigenetic regulators may affect an array of biological processes/hallmarks of cancer or, instead, have greater bearing on one major biochemical pathway contributing to tumorigenesis. TFs such as the AR and FOXA1 and epigenetic modulators such as EZH2 have certainly been shown to be pleiotropic, directly affecting multiple biological functions and, as such, producing multiple phenotypic changes. On the other hand, TFs such as E2F, SOX9, TWIST1, and ASCL1 affect single (albeit central to survival and growth) processes such as proliferation, differentiation, and lineage determination. A short summary of TFs important in PCa pathophysiology can be seen in Figure 1.

The importance of TFs (and the AR in particular) in PCa can be illustrated by (1) its effectiveness as a therapeutic target, (2) its activity as a prognostic biomarker, and (3) the effect of its targeting on the evolution of PCa.

Upon androgen (dihydrotestosterone; DHT) stimulation, the AR regulates gene transcription through crosstalk with other transcription factors, nuclear translocation, and binding to androgen response elements to regulate prostate development [11]. Transcriptional cross-talk occurs with a range of other factors including ETS TFs whose expression is aberrantly upregulated through chromosomal translocation and amplification, c-Myc, and FOXA1/Forkhead factors [12]. Despite this intricate biological complexity, the PCa field has predominantly focused on the AR in isolation for the derivation of activity-based gene signatures to predict whether individual cancers are AR-dependent or not. This is the case predominantly for historic reasons, as prior to the identification of the AR, Charles Huggins showed that castration could restrict PCa growth [13], thus leading to the discovery and characterization of the AR long before other relevant markers. This approach, however, may have led to an oversimplification of the disease and a focus on AR target genes associated with the androgen response [14].

The AR is most highly expressed in luminal epithelial cells, which produce prostate-specific antigen (PSA), a major diagnostic biomarker in PCa. Its transcriptional activity supports a specialized differentiation function that comprises glycolytic and anabolic metabolism, glycosylation, calcium and lipid metabolism, and protein folding. This is achieved by either direct binding or proximity effects on neighboring genes, leading to the transformed prostate observed in localized PCa. AR activity is frequently sustained as PCa progresses and the repertoire of binding sites and target genes evolves. Consequently, androgen deprivation therapy (ADT) with agents such as enzalutamide and abiraterone has been exceptionally effective in 80% of PCa cases [15].

Predicting the differentiation potential of PCa tumors is important because some PCa cells survive and display plasticity, which is the ability of cells to adapt to extrinsic and intrinsic perturbations and differentiate from a stem-like to a differentiated-like phenotype permanently or be in constant flux between the two (de-differentiation). Indeed, it has been shown through the transcriptional profiling of epithelial tissue that aggressive PCa is enriched for a prostate basal stem cell signature, particularly for E2F cell cycle target genes [16]. Such dramatic changes in cell morphology are accompanied by stark shifts in transcriptional activity and lead to PCa that is ADT- or castration-resistant (CRPC) through mechanisms that include non-canonical androgen production and AR gene and/or protein upregulation. This shift towards aggressive forms of PCa has become more apparent as AR-targeted therapies have become more effective, particularly in perturbing the hormone-dependent activation of the AR. In these cases, other TFs are known to be particularly dysregulated including RB-loss, p53-loss, and E2F activation. These CRPC cases are characterized by various types of treatment or intrinsic biology-induced differentiation that lead to lethal small cell/neuroendocrine (NEPC), intermediate atypical (IAC), or even double negative PCa (DNPC), which displays a lack of AR/PSA and neuroendocrine markers. These dynamic states propose challenges for the assessment of gene expression and associated clinical predictions based on single samples.

Given the central role of the AR in prostate cancer tumorigenesis, extensive effort has been put into developing activity gene signatures for this factor. However, the AR does not act alone. p53 and HIF1α are believed to have a significant impact on prostate cancer prognosis [17,18]. More recently, TFs that are important in cellular homeostasis and normal cell physiology such as the unfolded protein response transducer XBP1 has been identified as a potential key driver of PCa progression [19,20]. Gene activity signatures are predominantly centered around two distinct categories of master regulators: epigenetic regulators and TFs. In PCa, signatures have been generated for epigenetic modifiers such as BET and EZH2, as well as for TFs such as the AR. EZH2, for example, has been associated with metastatic PCa development post-radiotherapy [21], correlated with genomic drivers of PCa progression such as PTEN [22], and found to cooperate with BRCA1 to maintain cancer stem cell populations in PCa [23]. In addition, EZH2 inhibition was shown to reverse anti-androgen resistance in murine and human models of PCa that exhibited increased lineage plasticity, metastatic potential, and treatment resistance due to RB-1 and p53 loss [24].

As lethal types of PCa become more prevalent, the search for TFs alongside the AR responsible for PCa progression has intensified, and there is no shortage of candidates as nuclear hormone receptors include the glucocorticoid, estrogen, and progesterone receptors, the latter being also of paramount importance in breast cancer and other metabolically active malignancies. The glucocorticoid receptor (GR) for example, was shown to bypass the AR blockade and confer treatment resistance in the LNCaP/AR Resistant to Enzalutamide Xenograft derived (LREX’) in vivo model [25]. GATA2 regulates the AR and coordinates with FOXA1 to promote AR signaling, and GLI2 interacts with the AR to promote the hedgehog pathway to induce differentiation [12,26]. Helix–loop–helix (HLH) TFs such as ASCL1 are markedly upregulated in neuroendocrine ADT PCa models, and HIF1α may be a driver of a neuroendocrine phenotype under hypoxic conditions on top of being a universally accepted marker of aggressive neoplastic phenotypes. Other TFs that play important roles in regulating the lineage characteristics and differentiation status of PCa include SOX9 (a high mobility domain (HMG) TF) and other HLH TFs such as TWIST1, which enhances metastasis [27]. Another SOX family TF, SOX2, was shown to be upregulated in p53/RB-1-deficient PCa experimental models exhibiting traits of lineage plasticity. This upregulation was abrogated upon restoration of p53/RB-1 function [28]. Similarly, SOX11 was associated with regions of high-proliferative, neuroendocrine potential in genetically engineered murine PCa models [29]. Expanding on the theme of neuroendocrine differentiation, BRN2 transcription and, as a consequence, SOX2 regulation were shown to be directly repressed by the AR, thus contributing to an AR-indifferent neuroendocrine PCa phenotype [30]. TFs such as RUNX2 can drive endothelial mesenchymal transition (EMT), and MYC may impact WNT signaling and hence differentiation in PCa while also impacting homeostasis and proteostasis through the unfolded protein response (UPR) impacting treatment resistance and differentiation maintenance. An interesting combinatorial approach showcased the importance of MYC-dependent alternative pre-mRNA splicing through an rMATS-turbo large scale splicing analysis and a subsequent pathway enrichment activity study (PEGASAS) correlating the transcriptional signatures of 50 driver pathways [27]. Given this biological complexity, how does one then tailor transcriptomic signatures that may be used to more effectively classify diseases? What information is needed, and what pre-clinical models can provide the basis for such analysis?

### 2.1. Transcription Factor Centred Cignatures: Reverse vs Forward Translation

As discussed earlier the signatures with most potential would be ones covering a pleiotropic, multi-phenotype biology. Developing such signatures requires appropriately sized clinical sample groups for statistical power (reverse translation) and to accommodate disease heterogeneity. Resolving heterogeneity and achieving statistical power is very challenging, particularly when molecular data are generated from bulk samples. An alternative strategy is to initiate signature development using pre-clinical models. In this scenario, signature derivation is comparatively simple (forward translation), but the translation of such signatures into the clinic is much harder due to the complexity of clinical samples. This discrepancy is due to the fact that most pre-clinical models tend to be reductionist. Progress is being made to bridge this gap with recent advances in tissue explant and organoid culture, retaining some clinical complexity in a pre-clinical setting [31,32]. Signatures derived through reverse translation do not cleanly represent the activity of any single transcription factor. By contrast, TF activity signatures are almost always examples of “forward translation,” with an exception being PSA. The clinical observation that castration can restrict prostate cancer development and the development of PSA as a clinical biomarker both reflect AR activity. Single markers such as PSA do not, however, account for the levels of complexity the TF regulatory networks possess and crucially do not account for the impact of therapeutics targeting TFs in disease progression and treatment resistance development. Functional activity signatures provide more granular information regarding how these TFs affect cancer cell biology both pre- and post-treatment. A summary of the activity signatures generated in PCa oncology, both commercially and in academic medical research, can be found in Table 1.

### 2.2. Chromatin Landscape

To derive robust activity signatures, one cannot ignore the breadth of information provided by the chromatin landscape; rather, a multi-layered approach is needed. Such an approach needs to be highly sensitive and applicable to profiling chromatin landscapes in low cell numbers to discern in more granularity the different roles that TFs play in heterogeneous populations in situ. Exploring the chromatin architecture post treatment could uncover a wealth of information that could guide decisions as to whether changes seen at the transcriptome level through RNA sequencing are regulated by TFs—information that ChiP sequencing can provide. The pharmacological modulation of the AR may alter chromatin opening and, as such, the downstream effects seen as a result of corresponding therapeutic regimes. New versions of prognostic and diagnostic activity signatures should accommodate for the dynamic change of chromatin architecture upon pharmacological modulation and harvest the potential of combinatorial omics analysis, combining different types of sequencing outputs. Assays for Transposase-Accessible Chromatin using sequencing (ATAC) for example, offers an added component to an integrated epigenomic analysis to identify signatures of active chromatin. In addition, ATAC is amenable to single cell integration, making spatial- and cell-specific analyses possible.

There are several approaches to identify the TFs that are the most relevant regulators of a gene signature. This often entails the integration ChIP-seq and gene expression datasets to identify true regulatory (TREG) TF–gene interactions in terms of interaction probability aiding in the generation of complex disease transcriptional profiles [49]. Prostate cell-specific signatures were recently generated through a discriminant function analysis, accompanying the identification of a subset of primary PCa tumors with a low luminal epithelial enrichment and a high mutational burden to assess the degree of differentiation of luminal and basal epithelial cells in primary and metastatic PCa tissue samples [50].

Consequently, integrating the chromatin landscape architecture and transcriptomic landscape may prove critical in inferring regulatory relationships in clinical studies. This will occur because the accessibility of the chromatin will be variable in certain conditions, and transcript expression may provide useful information as to the TFs responsible for driving PCa progression. Changes in chromatin accessibility alter the binding ability of TFs playing a significant role in CRPC because AR-regulated, bromodomain-containing proteins such as BRD2 mediate chromatin relaxation, thus leading to the identification of a bromodomain-related gene stratification signature, BROMO-10 [41]. Integrative analyses in lung and brain metastatic cells of breast origin utilizing ATAC-seq TCGA data, ChIP-seq, and chromosome conformation capture showed distinct chromatin accessibility signatures (metATAC) of active chromatin, identifying TFs such as TFAP2C as markers of metastatic progression [51]. Chromatin dysregulation studies have uncovered important regulators of TF activity such as the chromatin remodeler gene CHD1. A study combining ATAC-seq, RNAseq, and CRISPR screening established that the loss of CHD1 promotes a PCa phenotype of lineage plasticity/chromatin dysregulation and decreased susceptibility to anti-androgens through the dysregulation of the TFs NR3C1, BRN2, NR2F1, and TBX2 [52]. Furthermore, ChIP-seq showed that deleting CHD1 re-organized the AR cistrome to an AR cistrome mimicking that of an oncogenic PCa phenotype, thus driving tumor growth in murine models of PCa through TFs such as HOXB13, BRN2, and GR [53]. A further epigenomic analysis involving the generation of 268 epigenomic datasets found that reprogrammed AR chromatin binding sites in PCa are usually occupied by FOXA1 and HOXB13 in the normal prostate, thus pointing to specific metastatic and lineage reprogramming roles for HOXB13, FOXA1, and NKX3-1 [54]. During integrative analysis in N-Myc biology, the transcriptome, cistrome, and interactome were investigated to reveal a neural lineage plasticity switch associated with epigenetic reprogramming [47]. N-Myc was shown to overlap with FOXA1 and HOXB13, as well as to facilitate reprograming of histone marks associated with lineage plasticity. Interestingly, EZH2 seemed to correlate with N-Myc, as the inhibition of EZH2 reversed the suppressive lineage effects of N-Myc induction. This work led to a signature derived from xenograft 22RV1 models of PCa that was further enriched by a stem/neuronal component [55]. N-Myc overexpression has been separately shown to drive PCa aggressiveness in pre-clinical models with reduced AR signaling by producing a phenotype in these pre-clinical models of poor differentiation and invasion [56]. Integrative gene signatures involving N-Myc have also been interrogated in neuroblastoma, where it was found to be in association with activity scores of chromosome 1p deletion, chromosome 11q deletion, and chromosome 11q whole loss to predict patient survival through multivariate Cox regression models [57]. Epigenetic silencing was deemed to be a feature of metastatic potential because negative regulators of WNT and growth signaling showed higher levels of methylation in a study using a combination of ontology and multivariate Cox regression and log-rank analysis to produce a metastatic assay [44] validated in biopsies to improve risk stratification in radically irradiated PCa [58]. Mapping genome-wide H3K27 methylation in aggressive cancer tissues and Oncomine-derived microarray data led to the generation of a polycomb repression signature of 14 direct targets of polycomb group proteins such as EZH2 [59]. As discussed earlier in this review, FOXA1 is an indispensable TF for differentiation through binding to enhancer sequences; indeed, it has been shown that E2-signaling epigenetic signatures are translated by FOXA1 to establish lineage-specific transcriptional programs [60]. Indeed, FOXA1 mutations have been classified into three structural classes conferring separate PCa prognostic characteristics: increased chromatin mobility in early PCa (class I activating mutations), WNT-driven PCa metastasis (class II activating mutations), and established metastatic PCa (class III genomic rearrangements) [61]. FOXA1 mutations also play a role in differentiation, as the ATAC-sec of wild type and mutant FOXA1 uncovered signatures of diverse mutational effects including luminal epithelial differentiation and mesenchymal/neuroendocrine reprogramming [62].

## 3. Integration of Multiple Datasets: Combining Forward and Reverse Translation

**Figure 2:**
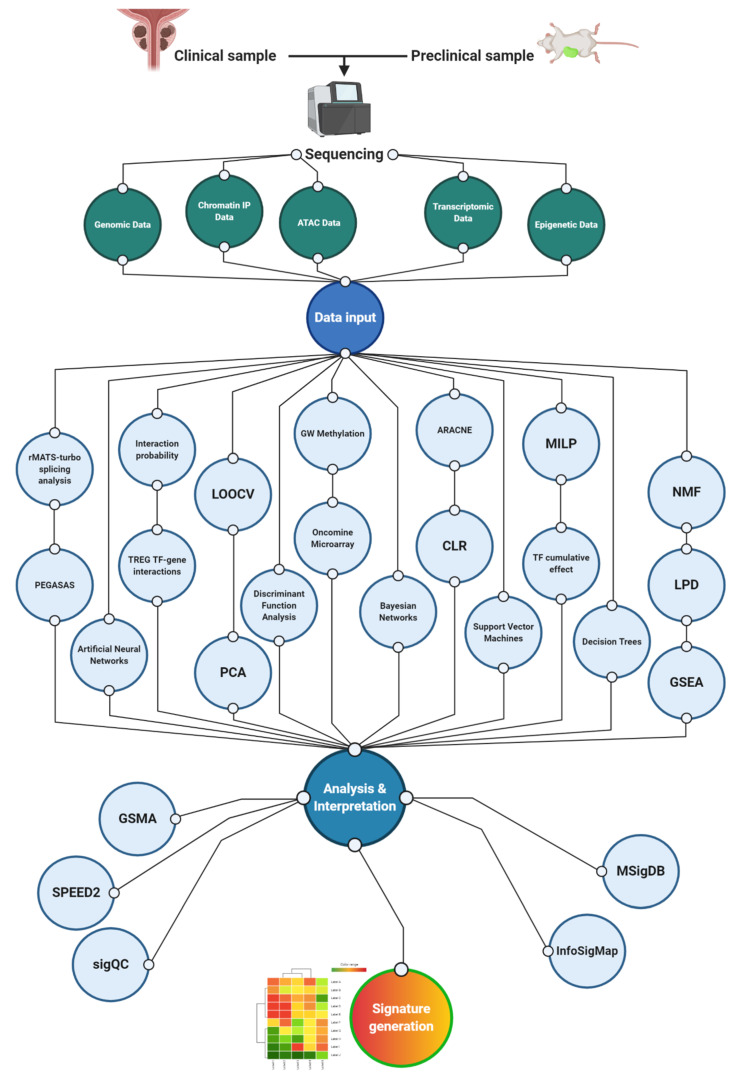
Overview of tools used in activity signature generation. A multitude of tools are used in generating activity signatures in cancer generally and in PCa particularly. These clusters are a representative, non-exhaustive range of tools used in PCa studies mentioned in this review. Consequently, they are forming workflows according to examples cited in this manuscript; that is not to say that the paths across these workflows are not routinely integrated to produce activity signatures. The main cluster represents analytics performed directly to dissect data for the purpose of signature generation. These take data from epigenetic, transcriptomic, Assays for Transposase-Accessible Chromatin using sequencing (ATAC), and genomic data as inputs and include calculating interaction probability scores between TFs and targets [49]; correlating transcriptional signatures of multiple driver pathways [27]; cross validating gene expression signatures using leave one out cross validation (LOOCV) and principal component analysis (PCA) plots [65]; assessing tumoral degree of differentiation through discriminant function analysis [50]; combining genome-wide methylation with microarray data to generate polycomb signatures [59]; and utilizing artificial neural networks, Bayesian networks, support vector machines and decision trees to combine existing data and generate cancer prediction models [66]. Moreover, they use mixed integer linear programming (MILP) to discern downstream cumulative effects of TFs [67] and incorporate nonnegative matrix factorization (NMF), latent Process Decomposition (LPD), and gene set enrichment (GSEA) analyses to combine signatures to attempt to generate combination signatures involving multiple biochemical pathways [43]. The cluster on the left-hand side of the figure represents tools that are developed to test signature reproducibility across heterogenous and numerous datasets including those analyzing multiple independent studies of the same condition through gene signature using meta-analysis (GSMA) [68], inferring signaling pathways affected by TFs (SPEED2) [69], and determining signature validity across datasets (SigQC) [70]. The cluster on the right-hand side of the figure represents signature maps and repositories where one can access multiple signatures for validation or further experimentation including the Molecular Signatures Database (MSigDB) [71] and InfoSigMap [72]. Acronyms used in the figure: LOOCV (leave-one-out cross-validation); PCA (principal component analysis); GW (genome-wide); ARACNE (Algorithm for the Reconstruction of Accurate Cellular Network); CLR (context likelihood of relatedness); MILP (mixed integer linear programming); NMF (nonnegative matrix factorization); LPD (Latent Process Decomposition); GSEA (gene set enrichment analysis); and GSMA (gene signature using meta-analysis). Created with BioRender.com.

The emergence of AR-indifferent CRPC has been partly attributed to the prevalence of constitutively active AR variants that lack the canonical ligand binding domain. As such, the presence of these variants, which include AR-V7, provides a barrier to effective androgen deprivation therapy [63]. The knowledge that AR-V7 may contribute to treatment-resistant phenotype emergence has led to clinical studies showing that it is rarely present in primary PCa but is prevalent in CRPC and enriched in patient groups after the administration of ADT (particularly enzalutamide/abiraterone), correlating with a CRPC 59-gene signature that includes HOXB13 [42]. This AR-centric approach has led to further clinical investigations based on molecular signatures consisting of nuclear AR-N overexpression, cytoplasmic CYP17 expression and a specified ratio of AR-C terminal/AR-N terminal domain expression [64]. These constitute examples of studies that have utilized both directions of the laboratory-to-clinic palindrome, incorporating the prognostic validation of TF-centric signatures within clinical trials. Such an approach highlights the complexity of TF signatures since one must consider multiple forms of the same TF, as well as a combination of multiple TFs. This complexity is reflected by the number of workflows and tools routinely used to derive activity signatures. A non-exhaustive summary of these can be seen in Figure 2.

The further integrative analytics of transcriptomic and clinical data has shown a tumor-suppressive activity of a microphthalmia-associated transcription factor (MITF), which directly affects the heat shock protein crystallin alpha B that is downregulated in PCa. This is of particular interest as an example of a TF–target combination study that leads to a PCa prognostic signature [45]. Such signature-generating studies offer potential insight into the further integration of established tumorigenic biologies. MITF is a member of the MYC gene family and interacts with beta catenin, which is part of the WNT signaling pathway, a biology linked to cancer stemness and reprogramming. In the presence of WNT signaling, beta catenin stabilizes and leads to the activation of specific target genes including c-Myc [46], the amplification of which is a well-documented PCa driver. The fact that MITF is an Myc family member shown to play a role in PCa progression may suggest that Myc-dependent activity signatures should extend beyond c-Myc and c-Myc amplification or, indeed, n-Myc and n-Myc amplification in NEPC. Further clues to this can be found in other cancer types. MITF has been heavily implicated in melanoma progression, and AR-positive melanoma patients were shown to have worse prognosis because the AR promotes cell invasion through an MITF-AXL signaling axis [46]. Post-translational modifications of TFs are not usually taken into account when trying to generate activity signatures [73]; instead, they are mostly based on the transcription levels of a TF to estimate its activity via tools like Algorithm for the Reconstruction of Accurate Cellular Network (ARACNE) [74] and context likelihood of relatedness (CLR) [75]. Mixed linear integer programming (MILP), however, has been used to show MITF biology as a validation model of cumulative effects of TFs on target genes [73]. The MITF–Myc balance is a representative example of a TF co-dependency that promotes poor prognosis through a signature derived by a logistic regression model using MYCN amplification as the response variable [57] that could be used in PCa.

### 3.1. Integrative Analytics: Models for Signature Generation

As defined earlier, neither forward nor reverse translation in isolation seem likely to be the optimal way of deriving an activity signature. Rather, a pertinent question is: where should these two approaches intersect? The simplest example of forward and reverse translation coming together in PCa is the Prolaris signature. This was inspired by the development of activity signatures in breast carcinoma and was based on an initial 126 cell cycle progression (CCP) genes (GEO database) that were tested against 96 FFPE tumor sections, leading to a final signature of 31 CCP genes with an adjusted CCP score converging on E2F TFs [76]. Other signatures such as Decipher—which encompasses 22 genes involved in cell proliferation, structure, immune modulation, CCP, and AR signaling—aim to predict PCa progression after treatment [33]. This signature does not point to a single driver and is, as such, more difficult to narrow down a particular target candidate for synthetic lethality, but it captures a wider variety of cancer hallmarks, potentially including inter-tumor heterogeneity, thus leading to more potent patient stratification. CCP- and E2F-based signatures could be argued to be a valid substitute for the diagnostic KI67 stain processes. In 4000 tissue cores, KI67 showed significant inter-tumor variation, associating with stage, invasion, and Gleason score [77]. Since KI67 is strongly associated with cell proliferation and cancer growth, activity signatures encompassing CCP markers could, if not substitute, complement histological diagnostic/prognostic procedures. Another approach of forward/reverse translation intersection is the combination of PSA (reverse) and TF activity signature (forward). A serum response factor (SRF)-dependent, androgen-responsive gene signature correlated with PSA failure, as defined by the presence of PSA post prostatectomy—thus indicating CRPC progression. This study showed that the proportion of SRF- and AR-dependent genes associated with PSA failure outcompete direct AR target genes [78]. Such E2F/CCP/AR combinatorial approaches unveil potential CRPC/NEPC-specific targets such as the placental gene PEG10 that promotes cell cycle progression in a p53-deficient background, thus promoting invasion through Snail expression [79].

### 3.2. Combining Signatures: Dealing with an Increasing Data Load

The idea that signatures derived through alternative processes may converge to common prioritized pathways is certainly an attractive one. Two sets of signatures (SIG-HES6 and SIG-DENT) were used to link overlapping hormone signaling (HES6-AR) [80], cell cycle progression (Prolaris) [36], and a molecular subgroup of patients (PCS1) derived by the non-negative matrix factorization (NNMF) of control pathways (SIG-HES) and overlapping DESNT diagnostic signature [81] plus a second NNMF signature PCS3 (SIG-DESNT) to show that aggressive cancer development may be dependent on overlapping common pathways between signatures [43]. Unsupervised mathematical approaches such as the Latent Process Decomposition (LPD) attempt to include intra-PCa-sample tissue heterogeneity to categorize PCa according to prognostic criteria [82]. Such variable approaches have led to an increasingly annotated and analyzed wealth of information in the form of expression profile datasets and associated analyses such as the DISC cohort [83]. Other gene set repositories have been created, one of the most used of which is the Molecular Signatures Database (MSigDB) [84]. However, as one would expect, a repository containing more than 10,000 gene sets is hampered by redundancy and heterogeneity between different techniques used to derive such signatures. Following traditional gene set enrichment analysis (GSEA) [85], focused differential gene expression attempts have been made to simplify and summarize the findings of MSigDB by identifying groups of similar gene sets and biological themes, refining and determining hallmark datasets, and independently validating final hallmark gene signatures [71]. In addition to trying to decipher the downstream effects of TF biology, as annotated through transcriptomic differential expression, there have been attempts to pinpoint upstream events that lead to TF changes. These include SPEED and SPEED2, which are web servers that infer signaling pathways that lead to the deregulation of genes affected by TF activity when presented with a list of differentially expressed genes [69].

### 3.3. Tools to Normalise Increasing Number of Activity Signatures

With the growing number of activity signatures being published there has been a drive to develop tools that predict signature utility across heterogeneous datasets and easily map the increasing documentation of generated signatures.

Meta-analytic approaches on big data collections been attempted to specifically derive TF biological information. A pipeline deriving TF activity signatures from 1056 cell lines and 9250 primary tumors combined consensus TF regulons and gene-wise expression data with unsupervised enrichment algorithms [86]. These functionally characterized TF mutations linked genomic aberrations in cancer drivers with TF dysregulation and proposed new markers of drug response [86]. This approach utilized in vivo ChIP-seq experiments, in silico TF binding site predictions, and manual curations. Carrying out meta-analyses on a huge number of signatures is hampered by the relative inability to carry out analytics at a large scale and the difficulty with intra-data heterogeneity. A study attempted to overcome this by combining gene level *p*-values through addCLT to produce a framework, gene signature using meta-analysis (GSMA), that analyses multiple independent studies of the same condition and provides a global list of differentially expressed genes along with meta-*p*-values upon leave-one-out (LOO) analysis [68].

The derivation of activity gene signatures requires the application of analytical methods to omics data which include artificial neural networks (ANNs), Bayesian networks (BNs), support vector machines (SVMs), and decision trees (DTs) [66]. These methods can be applied to determine a subset of genes or pathways (if ontologies are taken into consideration) within a particular dataset. Principal component analyses (PCAs) can be used to then summarize gene signatures in single scores [87] however, there are differences between how separate platforms generate signatures and scores, as ensemble analyses have shown [88]. Attempts have been made to address the issues faced with gene signatures when applied to heterogenous patient populations, such as the SigQC package that aims to determine if a given gene signature can be used across datasets and if, in a PCA manner, can be summarized in a single score [70]. Furthermore, tools to signal functional redundancies between signatures have been developed. One such tool is InfoSigMap, an interactive map that is composed of 962 informative signatures that have been singled out for their likelihood to be enriched in multiple comparative cancer studies and, as such, have been used to determine cross-relationships of such signatures as well as the visualization of omics data [72].

As it stands, there seems to be a lack of standard analytical frameworks for signature generation methods. Reproducibility is a major hurdle to forward translation, especially since most signatures born out of such methods may share some common basic tools but vary greatly in methodology and are subject to scrutiny for novelty to reach publication. As such, further work is needed to integrate the need for new ways to generate signatures (including machine learning and artificial intelligence) and the need for standardization necessary to bring an activity signature to the clinic.

## 4. Multi-Omics and Evolution

Ultimately, current AR activity signatures are limited to classical AR targets because the models used to derive such consensus signatures emerged from data prior to the administration of an AR antagonist. Though such signatures have proven exceptionally useful, they offer little in prognosticating the response of individual patients to AR inhibition (ADT). As such, exploring how the TF landscape responds to treatment is preferential to exploring what the steady state of the disease is. That is not to say that information at the point of diagnosis is not important. Indeed, exploring heterogeneity at the levels of chromatin architecture, and gene expression can support the selection of patients for radical treatment and serve as a platform for more fine-tuned investigations monitoring disease progression and treatment efficacy in biological fluids (if radical prostatectomy has been undertaken). These would involve circulating tumor cells (CTCs), exosomes and circulating free DNA (cfDNA). This approach adds the key component that diagnostic and prognostic tools and signatures lack at the moment: dynamic evolution. This, of course, will also lead to a more nuanced approach to heterogeneity because no single marker, TF, or activity signature will be appropriate for any patient for the duration of the disease. One would, however, expect to see oscillations between states captured by admixtures of activity profiles associated with classical TFs involved with PCa, such as Myc, HIF1α, and the AR.

### 4.1. Co-Activation Signatures

Perhaps the most optimal way of developing such synergistic activity signatures would be through a combination of functional studies and clinical data. Indeed, it has been exceptionally interesting to see the emergence of co-activation signatures in prostate cancer that not only focus on one of the classical TFs associated with PCa progression (AR, Myc, p53, and HIF1α) but rather on a combination of an established TF with a biological master regulator or an established TF with another oncogene. One such example is the META-16 gene signature that aims to prognosticate treatment response and time to metastasis in patients with prostate osteometastatic clinical presentations [70]. The co-activation of Myc and Ras was deemed significant in predicting metastatic potential of PCa. A clinical database (PROMOTE; PROstate Cancer Medically Optimized Genome-Enhanced ThErapy) was interrogated because most of the samples within it constituted bone PCa metastases [89]. Genome-wide correlation studies were performed and found 559 genes positively correlating with Myc expression, 93% of which correlated with Ras activation. Confronting this signature on established bone metastasis signatures let to the identification of a 16-gene signature that was based on a univariable Cox proportional hazard model based on 336 metastasis-free TCGA patients [90]. This analysis was also supported by single cell sequencing of bone metastatic versus primary prostate tumors.

**Figure 3:**
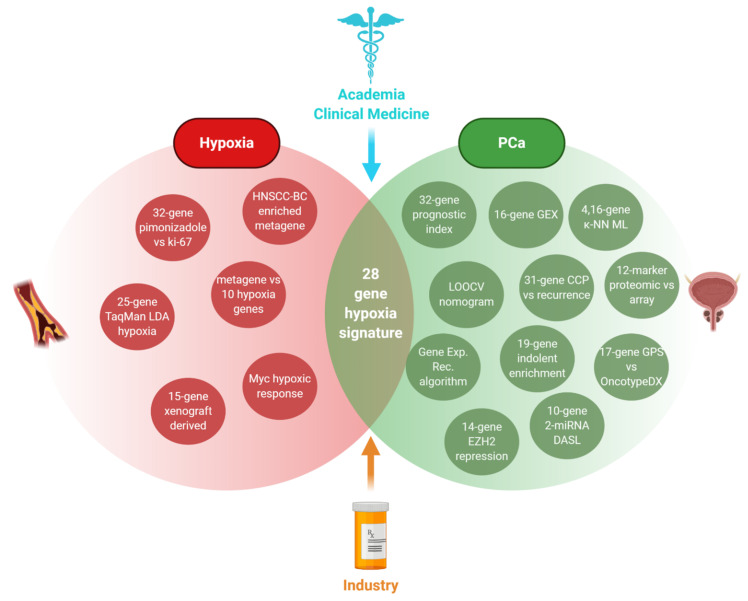
Composition of the 28 hypoxia gene signatures to prognosticate localized PCa and exemplify derivation of prognostic signatures by combining multiple sub-signatures [40]. The hypoxic component was comprised by (1) a 25-gene signature identified through TaqMan Low Density Array-Hypoxia Scores on tumor samples from 80 head and neck squamous cell carcinoma (HNSCC) patients [92], (2) a metagene enriched in hypoxia-regulated pathways through in vivo co-expression pattern data from head, neck, and breast cancer studies [93], (3) studies linking Myc to the modulation of the hypoxic response [94], (4) a 32-gene signature positively correlating to Ki-67 staining that was shown to link the hypoxia marker pimonidazole and aggressive PCa [95], (5) a 15 gene hypoxia-related expression classifier from in vivo xenograft tumors validated in 323 HNSCC patients [96], and (6) a metagene obtained by analysis of genes whose in vivo expression clustered with the expression of 10 well-known hypoxia-regulated genes [97]. Created with BioRender.com.

An alternative manner of crossing biological pathways is through the crossing of generalized biochemical responses rather than single TF signatures (e.g., HIF1α vs hypoxia and PCa vs AR) via both in vitro and in vivo data. Mining both hypoxia- and PCa-related signatures is how a 28-gene hypoxia-related prognostic signature was developed for localized PCa [40] (Figure 3). To illustrate the importance of hypoxia as an accompanying factor to carcinogenesis, a study quantified more than 8000 tumors in 19 tumor types to showcase the propensity of hypoxia for selecting for TP53 mutant cells to downregulate DNA repair genes and to correlate with other PCa drivers such as PTEN loss [91].

Hypoxia and genomic instability may be common indices that pinpoint chances of biochemical relapse, as identified by multivariate analyses that combined DNA-based and intra-prostatic hypoxia indices to predict patient stratification into treatment intensification trials [98]. This supports the premise of hypoxia as a driving force of genomic instability through the inhibition of recombination-mediated DNA double strand breaks [99].

### 4.2. Mutational Burden

Co-activation signatures pose an interesting question regarding the interplay between differential biochemical pathways that may determine the clinical relevance of basic biological research and the utility of TF activity signatures in PCa clinical practice. Contributing factors to be taken into consideration include driver mutational events linked to PCa pathogenesis. The caveat here is that the more signatures are generated on the back of further intersecting biologies, the more complicated the picture becomes, thus adding potentially valuable information for the clinic. For instance, one would expect a proportion of any signatures generated in PCa to reflect metabolic changes given that we know that metabolic change is a significant underpinning hallmark of cancer. In turn, we would expect those changes to reflect the stress response status of the tumor and, therefore, another feature of its evolution: the ability to accumulate DNA damage. This ability to ride the wave of DNA damage through an enhanced DNA damage response (DDR) and stress adaptation enables the tumor to survive and acquire somatic driver mutations. Such mutations include alterations in PTEN, p53, and RB-1. Targeting the DDR alongside the AR has recapitulated the potential of synthetic lethality in cancer treatment [100] (Table 2). Transcriptional profiling studies to produce DDR assays prognostic of survival in esophageal adenocarcinoma have pointed to the utility of further investigating pathways intertwined with the AR during carcinogenesis such as immune infiltration, brought about by STING activation [101]. Underscoring a conserved biology linked to responses to genomic instability, a 44 gene expression signature termed the DDR deficiency assay characterized in breast cancer [102], was applied to predict resistance of PCa to docetaxel [103]. AR loss has been shown to also be important in melanoma, where its downregulation lead to STING activation and dsDNA breaks as the AR anchors DNA repair the Ku70/80 proteins to RNA polymerase [104].

The identification of driver mutations has also enabled driver-centric transcriptional investigations into the effect of non-coding RNA in PCa progression, a field that opens new possibilities in studying the regulation of tumorigenesis, as TFs may regulate miRNA expression that, in turn, regulates the transcriptional repertoire of tumor growth through epigenetic regulation. For example, a network analysis of TP53-regulated transcriptional events has revealed that a tumor suppressor signature of long non-coding RNAs affects genes such as CDKN1A, BAX and BBC3 [111]. The role of RNAs, and miRNAs in particular, was investigated in PCa through DASL expression profiling that highlighted 10 protein coding genes and two miRNA genes including TFs such as NOTCH to predict PCa recurrence following radical prostatectomy [112].

This context provides a great opportunity to combine mutational data, activity signatures, non-coding RNA, and metabolomics proteomic data to produce a biomarker discovery pipeline that covers all bases of cancer evolution from the driver mutations to the adaptive mechanisms that enable cancer cells to thrive in such environments. The NMF of mutations has been used to model mutational signatures from mutational catalogues [113]. Furthermore, there is value in gathering information to discern tissue-specific and cancer-specific effects. This is because there is a distinction between mutational signatures linked to fundamental driver events (PTEN loss and TP53 loss) and mutational signatures that are linked to microenvironmental fluctuations, as the former points to potential evolution and the latter points to adaptation, both important components in accurate prognostication. Studies in primary cancers other than PCa provide tissue-specific tumorigenic changes that can point to similarities between PCa and other primary tumors, as well as provide clues as to how to derive clinically relevant TF signatures. For example, a nine TF signature derived from the breast cancer TCGA dataset closely recapitulated recurrence-free survival through univariate Cox proportional hazard analysis, least absolute shrinkage and selection operator (LASSO) Cox regression analysis, and multivariate Cox proportional hazard analysis [114].

Depending on the aggressiveness of these primary tumors, one can glean information as to what prognostic outcome of each PCa comparative tumor will be. Moreover, the analysis of primary tumors originating in likely sites of CRPC metastasis can provide clues to the molecular changes that need to occur for metastatic PCa to migrate to these sites. In addition, it may offer possibilities of combining work on key TF and stress signaling pathways to combine activity signatures to cover multiple key biochemical pathways in PCa progression. For example, a 38 hub gene signature was generated to stratify glioblastoma patients according to their IRE1/XBP1 status [115]. XBP1s is a TF and effector of the UPR, a mechanism that is triggered both by misfolded proteins due to somatic mutation (p53, ATM, etc.) and the accumulation of protein production demand due to the increase of neoplastic metabolic activity. At the same time, it has been shown that XBP1 is linked to both AR and Myc amplification. Such combinations of investigations would address the genomic, transcriptomic, and proteomic burden of cancer driver and maintenance events to distil the activity signatures of markers that predict disease progression and predict treatment susceptibility.

## 5. Conclusions and Future Perspectives

Activity signatures are the result of collaborative research bringing together pre-clinical in vitro, in vivo, and clinical data to intertwine genetic and epigenetic activity to confront biological reality and assess clinical translation. As their primary purpose is to be a clinical tool of prognostication and stratification, they are inherently limited by inter-patient and inter-tumoral heterogeneity. As such, a major problem with pre-clinical data-derived TF activity signatures would be the overextrapolation of translational value, which could be overcome by developing patient-derived models such as organoids or explants. In addition, there is a need for optimal conditions to assess if a signature is statistically clinically relevant. Consequently, the sample size (i.e., number of patients in any given study) and the condition these patients are in play significant roles in determining how representative and translational each signature is from a population and public health perspective. The explosion of the genomic era has led to many landmark discoveries in oncology and other fields. At the same time, though, it has flooded the field with information and gene signatures that not always represent a particular subset of patients but rather biological mechanisms that are found to be key in pre-clinical models. Moreover, the mechanisms that signatures are derived by may yield false positive results and poor specificity, particularly in complex biological processes, as described in a study comparing data-derived gene sets (signatures) against GO terms and literature-derived gene sets in immunological biological processes [116]. In parallel, there are signatures that are solely derived from observational clinical data that themselves are only based on tissue acquired at diagnosis. This approach is faced with the limitation that it ignores disease evolution and provides information to but a snapshot of the genomic/transcriptomic/proteomic/chromatin landscape in the lifetime of a tumor.

### 5.1. Beyond TFs

It thus logically follows that we not only need combinatorial approaches to activity signatures but also models of disease that allow us to study the temporal dynamics of disease progression (and genetic/epigenetic flux occurring in parallel), as well as diagnostic tests that can be acquired and bio-banked throughout the duration of disease evolution. After all, a significant issue with publicly available PCa patient databases is that they are reliant on survival as a primary determinant of disease progression. As such, it is difficult to determine whether an activity signature is representative and predictive of disease evolution if there are not enough survival data to support differential survival events (i.e., life/death leading to a Kaplan–Meier curve). Biochemical recurrence (BCR) is currently mainly predicted based on clinical observations. However, BCR-predictive signatures that include genes directly affecting TF activity (TRIM14 and SSTR1) have recently been generated using LASSO following high throughput screening combined with a risk and predictive value evaluation through a multivariate Cox regression analysis and time-dependent receiver operating characteristics [117]. An interesting intersection of data would include the consideration of longevity signatures with PCa-centered ones. Hepatic gene signatures have recently been associated with lifespan in pre-clinical models relating to oxidative phosphorylation, chronic hypoxia, drug metabolism, and palmitoylation, all while utilizing GENtervention, a model used to associate gene changes with increased survival [118].

### 5.2. Thinking about Signature Validity and Dynamic Disease Progression

Regarding the combinatorial approaches, it may well be pertinent to ask the question: of all the numerous signatures produced via different regulatory approaches in PCa and similar malignancies, how many actually converge to a common mixture of biochemical pathways and markers? Furthermore, what would the utility of a combined “master” signature be? In the end, how do we determine which signature is most clinically relevant? Currently, clonal selection is used as a way of determining if a marker is a driver of PCa. Consequently, the mutations identified through clonal selection are the ones selected for in research. However, if the observed biology is tied to a particular therapy and not a genetic driver, from a clinical perspective, it is less pertinent to know that the observed response is linked to a driver biology. Therefore, although underpinning driver mutations should be taken into consideration for prognostic reasons, determining the therapeutic regime from a pharmacological response activity signature does not have to be relevant to a driver biology. Indeed, pre-clinical models may become complementary to signature research but focus more on therapy selection rather than constitute the core pathway for signature development. This may be supported by the increasing availability of patient samples, which will reduce the dependency on cell lines. This, of course, may lead to translational activity becoming dependent on resources, with fewer centers being able to benefit and carry out such research. Of course, as discussed earlier in this review, mutational signatures can point to both driver events and micro-environmental changes. This is a direct reflection of a “concrete” mutational landscape against the dynamic character of cancer evolution in the context of its surroundings. The increasing emergence of mutational signatures may provide more translation into clinical practice, as mutational signatures provide cell-specific or environment-specific components. As such, spatial information may well form the next generation of techniques of validating activity signatures for specific populations within a tumor because it will be able to distinguish between cell-specific, tissue-specific, and lineage-specific events [119].

Combining pharmacologically-driven and genetically-driven signatures may provide more insight into the adaptation versus selection mechanisms responsible for disease relapse and evolution in treatment-resistant forms such as the PCa to CRPC conversion. This was exemplified by the Dunning R-3327-H adenocarcinoma that showed that disease progression was down to original tumor heterogeneity rather than an original pool of identical cancer cells that managed to adapt to treatment and eventually proliferate [120]. This, of course, is true but the presence of immune cells, fibroblasts, and lineage plastic cells adds an extra dimension on the potential of a tumor to become AR-indifferent and evolve into CRPC. It has been shown that the AR may repress transcriptional programs, thus leading to cancer-associated fibroblast (CAF) activation as a negative regulator of CAF effector genes including IL6, POSTN and MMP3 [121].

Regarding the dynamics of disease progression, it would be pertinent to look at sample availability for metastatic disease and study both the primary and metastatic samples for DNA mutations. The presence of somatic mutations in both primary and metastatic tumours from the same patient may be regarded as a evidence of clonal selection and provide insights into how PCa tumors evolve to survive treatment and disseminate. Anchoring activity signatures back to a genetic context may be an important way of determining what is valuable or not. The backdrop of this context may be tissue specificity. Crossing information from relevant comparisons, such as primary tumors vs metastatic tumors, prostatic tissue vs urine, and cancer cells vs immune cells vs fibroblasts, will give a “whole organism” picture of what constitutes the framework of disease progression. From this cross referencing, one could determine consensus signatures that are identifiable in easily accessible biological samples (e.g., blood and urine) rather than samples that require invasive procedures and consequent trauma (punch biopsies) or invasive procedures that do not offer any information about the local dynamic evolution of the disease (complete prostatectomies). Such an attempt to infer temporal sequence of events in carcinogenesis has been realized through the integration of somatic mutation and gene expression data using a mixed integer linear program in breast cancer. This formulation uses both simulated and TCGA data to pinpoint mutational events that produce gene expression events over time [67].

### 5.3. Attribution of Activity Weight Within a Signature

One may have to also consider the definition of an activity signature, like whether it is only the differential expression of prioritized genes within a dataset or that differential expression is normalized according to biological weight. A weighted signature will look completely different to one based solely on gene expression levels. Furthermore, if only gene expression levels are considered, then the signature’s applicability on a heterogenous clinical sample group is less likely to be valid. The rate limiting step in such an approach would be the threshold set that determines the weight of each gene (and, as such, its differential expression) within the signature. The threshold itself would also determine the number of patients that the signature would be applicable to. The less precise or constrained the threshold, the less selective the signature would be for patients potentially susceptible to the biologies (and hence treatments) that the signature is describing. On the other hand, if the threshold is over-constrained, it might miss patients that could be responsive to the represented biologies and treatments.

This points to the likely realization that PCa and other clinical entities will most benefit from signatures that are parts of gradients of tumoral activity. Crucially, the weighted component of each signature will be an identifier and measure of activity of where it is in the gradient, as each gene element would be weighed very precisely to measure susceptibility in a very constraint number of patients (in order not to miss patients). To this effect, bio-banking is a crucial and rate limiting component of such investigations.

### 5.4. Biobanking and Logistical Considerations

**Figure 4:**
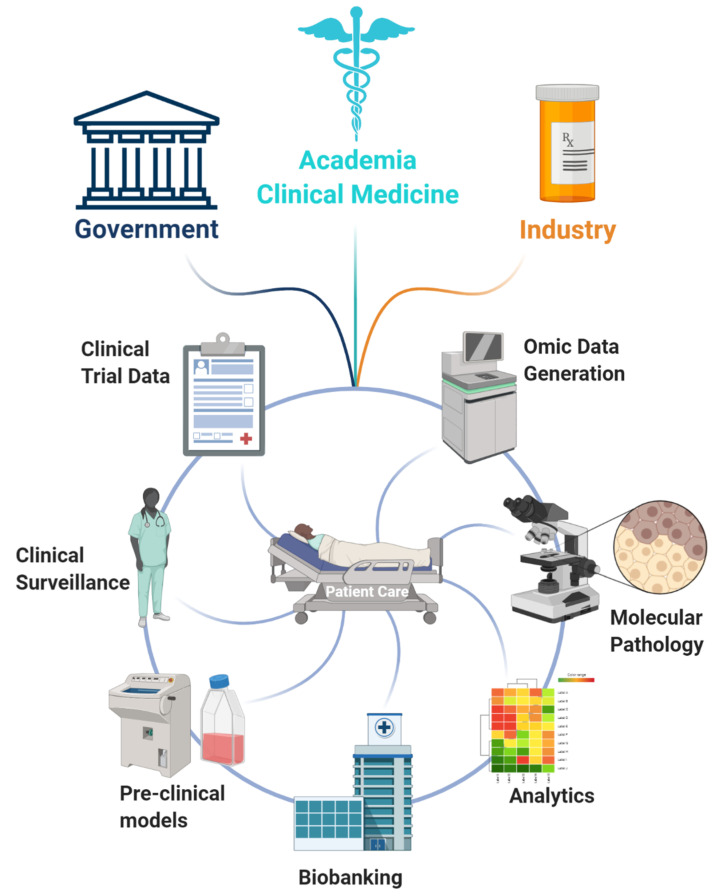
Main stakeholders and players in forward and reverse translation. Translation of activity signatures into clinical practice requires the integration of multiple data types and expertise. These include clinical trial data, clinical surveillance, humanized pre-clinical models, biobanks, integrative and extensive analytics, molecular pathology, and mass omics data generation. These need to be supported by public health bodies, industry shareholders, and academic researchers to achieve a harmonious, streamlined workflow of data integration culminating in multidisciplinary clinical care and personalized medicine. These collaborations are exemplified by programs such as (IMPRESS, Oslo, Norway) [122] and (CONNECT, Norway) and pan European educational drives such as BioS [125]. Created with BioRender.com.

The creation and maintenance of biobanks is a laborious and expensive process. This unfortunately does not make it possible for most research institutes and clinics across the globe. Ideally, the maintenance of biobanks would be under multi-institutional or national control with multiple stakeholders including public bodies (health governance and government) and private companies (Figure 4). Examples of such collaborations have been recently been set up in Norway with the IMPRESS [122] and CONNECT initiatives trying to build a national precision oncology framework involving private and public funding. Such infrastructures further attract multinational collaborations, as exemplified by the recent prioritization of collaboration by the EMBL with Nordic countries. A study characterizing 1577 primary PCas into molecular subtypes [123] is another example of large validation studies being undertaken through large scale partnerships to provide pertinent information to clinicians and patients alike [124]. There is, of course, an alternative model with single major hospitals driving omics signatures and stratification such as the Tumour Profiling Unit at the Institute for Cancer Research London, UK at the Royal Marsden and the Precision Medicine Centre of Excellence QUB in Belfast, UK.

Moreover, stochastic data accumulation from biobanks would require a dedicated funding stream to maintain and curate data libraries based on samples routinely collected from multiple clinical presentations and patients without a particular research question in mind other than accumulating data for potential future promising research. Of course, this once again poses a barrier to true data sharing because it asks the question of ownership. If only a handful of institutions can finance such ventures, then the risk/benefit ratio of investing in one to produce clinically relevant signatures becomes a lot more complicated because the utility of signatures in the clinic is not as regulated as the utilization of pharmaceuticals. Indeed, it may well be down to individual clinicians based on their research expertise and collaborations to determine if signatures would be a valid diagnostic/prognostic support tool in the analysis of the genomic/transcriptomic data of their patients to design individualized treatments. This poses the very real prospect of a whole new generation of clinicians needing to be trained in the art of interpreting omics data, as exemplified by EU/ERASMUS+-driven projects such as BioS that aim to train health professionals in the utility of bioinformatics and data analytics [125].

## Figures and Tables

**Table 1:** Signatures involving TF biology in PCa. There have been significant advances in molecularly subtyping PCa with a few diagnostic and prognostic tests available [33]. However, the widespread clinical utility of such tests in PCa is still being reviewed, with the complex underlying biology and disease heterogeneity posing significant barriers to a population-wide utilization. Moreover, most commercially available signatures were the products of reverse translation and clinical profiling. This is because the application of these signatures is in an incredibly complex setting (the clinic), and so far, there have not been sufficiently powerful pre-clinical models to overcome issues such as heterogeneity. This is increasingly being addressed by the emergence of patient-derived explants and organoids, which offer a significantly improved translational model of PCa compared to cell lines. As this table demonstrates, there is a drive in pre-clinical research to combine signatures to determine the effect of multiple key molecular pathways on disease evolution. This is a space that most commercially available signatures are currently not occupying as they focus on singular TFs (androgen receptor; AR) or single biologies (cell cycle progression; CCP). The further commercial and clinical validation of such approaches is of course needed, and this will be aided by the development of academic–government–industry collaborations and extensive biobank-databank repositories. MITF: microphthalmia-associated transcription factor; CPRC: castration-resistant prostate cancer.

Scheme	Signature	Mechanism	Translation	Ref.
Commercial	Oncotype DX	12 genes related to androgen metabolism, cellular organization, proliferation, and stromal response plus 5 reference genes	Reverse	[34,35]
	Prolaris	31 cell cycle genes	Reverse	[36]
	Decipher	22 genes involved in proliferation, structure, immune modulation, cell cycle and androgen signaling	Reverse	[37]
	ExoDX	Urinary-derived exosomal gene signature based on PCA3 and ERG RNA levels	Reverse	[38]
	SelectMDx	2 gene signature (DLX1 and HOXC6)	Reverse	[39]
Academic	Hypoxia-28	6 hypoxia and 11 PCa signature overlap	Combinatorial	[40]
	BROMO-10	10 gene signature from bromodomain inhibitor treated cells and PCa cohorts	Forward	[41]
	AR-v7	Variant correlation with 59 gene CRPC signature	Reverse	[42]
	SIG-DENT SIG-HES6	Overlapping PCa-related signatures	Combinatorial	[43]
	Primary PCa metastatic assay	70 gene signature to predict risk of biochemical recurrence	Reverse	[44]
	AR–MITF–Myc	Combination of signatures to prognosticate PCa survival	Forward	[45,46]
	MycN-EZH2	Transcriptome/cistrome/interactome N-Myc-derived signature in advanced PCa	Forward	[47]
	mCRPC-26	Treatment-resistant CRPC classification	Combinatorial	[48]

**Table 2:** Examples of (DNA damage response) DDR synthetic lethality in PCa. TFs in clinical practice from a treatment perspective. Stratification of patients in clinical trials aligns to treatment and relies mostly on mutational classifiers rather than activity signatures. TFs such as MYC, TP53, ATM, ATR, PARP1, BRACA1/2, DNA-PK, and GATA2 have been targeted through rucaparib (PARP1-rucaparib/enzalutamide/abiraterone), berzosertib (ATR-berzosertib/carboplatin/docetaxel) and CC-115 (DNA-PK; CC-115/enzalutamide) in current clinical trials [105].

Table	Therapeutic Combination	Clinical Trial Identifier	Ref
PARP-1	Rucaparib/enzalutamide	NCT04455750	[106]
PARP-1	Veliparib/abiraterone	NCT01576172	[107]
ATR	Berzosertib/carboplatin/docetaxel	NCT03517969	[108]
DNA-PK	CC-115/enzalutamide	NCT02833883	[109]
ATM	AZD0156/FOLFIRI	NCT02588105	[110]
